# Person-centred suicide prevention: key elements from the perspective of people living with suicidality

**DOI:** 10.1080/17482631.2025.2549752

**Published:** 2025-08-28

**Authors:** Malin Rex, Margda Waern, Eric Carlström, Isabelle Joneken, Susanne Tell, Thomas Brezicka, Lilas Ali

**Affiliations:** a Institute of Health and Care Sciences, Sahlgrenska Academy Gothenburg, University of Gothenburg, Gothenburg, Sweden; b Department of Affective Disorders, Sahlgrenska University Hospital, Gothenburg, Sweden; c Centre for Person-Centred Care (GPCC) Gothenburg, University of Gothenburg, Gothenburg, Sweden; d Institute of Neuroscience and Physiology, Sahlgrenska Academy Gothenburg, University of Gothenburg, Gothenburg, Sweden; e Department of Psychotic Disorders, Sahlgrenska University Hospital, Gothenburg, Sweden; f MIND Suicide Helpline, Stockholm, Sweden; g Centre for Person-Centred Care (GPCC) Person Council; h Department for Quality and Patient Safety, Sahlgrenska University Hospital, Gothenburg, Sweden

**Keywords:** Lived experience, mood disorders, patient safety, psychiatry, self-injurious behavior, suicide

## Abstract

**Purpose:**

The perspectives of individuals with lived experience are essential to understanding how care practices support or hinder person-centred suicide prevention. This study explores experiences of individuals who sought healthcare in Sweden during suicidal crises.

**Methods:**

In-depth interviews with 28 individuals with current or past suicidal behaviour were analysed using a phenomenological hermeneutical approach.

**Results:**

Six themes emerged: (1) Hoping for the best, while preparing for the worst, (2) The risks of help-seeking, (3) In need of a safe space, (4) Support from professionals, (5) “Now it’s your turn”, and (6) A shared journey. Findings indicate that individuals with suicidal behaviour view themselves as motivated and capable partners in shaping the care process and believe that co-creating care can help delay or lessen exacerbations. Participants who co-created their care felt better prepared for self-care during early escalation and more confident that, if their condition worsened, they and their healthcare team could address a shared challenge.

**Conclusion:**

According to participants, key elements of person-centred care include early engagement, long-term goals, and planning for crises. During intense suicidality, they emphasised the need for supportive environments and relationships—over mere security. Mutual trust between patient and healthcare team was seen as essential.

## Background

Suicidal ideation and behaviours affect millions of people annually (World Health Organization, 2021). While suicidality may manifest as a result of underlying mental health condition (Franklin et al., 2017), other factors—including family- and childhood related issues (Edwards et al., 2024; Luhaäär & Sisask, 2018; Moody et al., 2023; Zatti et al., 2017), existential concerns (Rydberg Sterner et al., 2020; Søberg et al., 2018, 2023; Van Orden et al., 2015), sociodemographic status (Messias et al., 2023; Schmidtke et al., 1996; Spataro et al., 2024), financial hardships (Sinyor et al., 2024; Naranjo et al., 2021), exposure to violence (Devries et al., 2013; Kim et al., 2022; Oltvolgyi et al., 2024; Pompili et al., 2013), substance use (Østergaard et al., 2017; Poorolajal et al., 2016), physical health concerns (Carbajal et al., 2017; Fässberg et al., 2016) and discrimination (Goodwill et al., 2021; Madubata et al., 2022; Wyman Battalen et al., 2021)—also play significant roles. The suicidal process typically develops over a period marked by psychological suffering, ultimately leading to a crisis aimed at relieving overwhelming distress (Buus et al., 2014; Hechinger & Fringer, 2021; Marcinkevičiūtė et al., 2024; Shamsaei et al., 2020). Individuals with lived experience of suicidality report experiencing mixed volitional feelings in the period leading up to an attempt (Pavulans et al., 2012), during the attempt itself (Savani & Gearing, 2023), as well as ambivalence towards seeking help (Blanchard & Farber, 2020; Hechinger & Fringer, 2021). The experience of existing on the border between being in control and losing control is consistently highlighted in previous studies (Berglund et al., 2016; Hagen et al., 2020; Pavulans et al., 2012). Many hesitate to disclose their thoughts to professionals due to fear of undesired consequences of disclosure or help-seeking (Blanchard & Farber, 2020; Finlayson-Short et al., 2020; Hechinger & Fringer, 2021; Krychiw & Ward-Ciesielski, 2019), or of being a burden to others (Krychiw & Ward-Ciesielski, 2019). Many also worry that care professionals cannot understand what it is like to be in a suicidal state (Maple et al., 2020). Being empathically treated and regarded as a resourceful individual promotes recovery (Hechinger & Fringer, 2021; Lindgren et al., 2011; Shand et al., 2018), whereas invalidation or indifference can impede it (Hechinger & Fringer, 2021; Krychiw & Ward-Ciesielski, 2019) or even elicit suicidality (Hed et al., 2024).

In high-income countries such as Sweden, where this study was conducted, clinical suicide prevention strategies have primarily focused on risk prediction in individuals seeking mental health support, based on statistical risk factors (Hawton et al., 2022). This emphasis has prompted regulatory authorities to criticize organizations for inadequately documented risk assessments (Hawton et al., 2022), leading to the prioritization of such assessments (Espeland et al., 2021; Hawton et al., 2022) and the establishment of routines specifically aimed at preventing incidents related to missed assessments (Fröding et al., 2021). Previous studies show healthcare professionals understand how static risk stratification fails to capture the dynamic nature of suicidality, which fluctuates over time, and how overemphasizing risk assessment may obscure the broader context (Espeland et al., 2021; Waern et al., 2016). Nevertheless, fear of a potential adverse events (Waern et al., 2016) and possible liability ultimately drives them to comply with these policies (Espeland et al., 2021).

Increasing recognition of the role that social determinants and somatic conditions play in influencing suicidality has led to findings underscoring the need to broaden the perspective on symptoms associated with suicidality (Asheim et al., 2024; Heinrich et al., 2022; Lofman et al., 2011; Pigeon et al., 2016; Pirkis et al., 2024; Sarchiapone et al., 2014; L. Smith et al., 2023). Within Swedish healthcare settings, those with long-term conditions and persistent or recurring suicidality are typically referred to mental health services (Fröding et al., 2021; Hadlaczky et al., 2012). While targeted suicide prevention measures, such as the Attempted Suicide Short Intervention Program (Gysin-Maillart & Michel, 2015) and the Collaborative Assessment and Management of Suicidality (Jobes, 2016), are emerging, their wide-spread adoption as standard clinical practices remain limited (Lindström et al., 2024). Current European treatment protocols often involve optimizing pharmacological approaches to manage underlying conditions, supplemented by psychoeducation and various psychotherapies (Hadlaczky et al., 2012; Wasserman et al., 2012). If detected and assessed by healthcare teams, acute life-threatening behaviour is typically treated in inpatient facilities. These are primarily staffed by physicians, nurses, and nursing assistants, the three most common professional groups (Gabrielsson et al., 2014).

In recent years, parts of suicidology have undergone a discursive shift, moving from viewing the patient primarily as a passive recipient of assessment and medical intervention to exploring the potential of a person-centred approach (Hawton et al., 2022; Watling et al., 2022). The latter emphasizes that the healthcare team and the patient work in a partnership based on a mutual agreement regarding treatment and recovery (Hawton et al., 2022; Lindström et al., 2024; Ryberg et al., 2020). Research on the development of services for people with complex mental health and social needs indicates that healthcare activities must be designed “with and for” the individual receiving care (Evans et al., 2023; Trevillion et al., 2022). However, previous studies indicate that frameworks based on shared decision-making models are rarely offered to patients with complex conditions in mental healthcare (De las Cuevas & Peñate, 2014; Drivenes et al., 2020; Haugom et al., 2022; Slade, 2017). The person-centred perspective, which emphasizes collaborative relationships among stakeholders, was initially developed as a concept for patient safety, primarily within somatic healthcare pathways, in the early 2000s (Institute of Medicine Committee on Quality of Health Care in A, 2001). Since then it been applied to the mental health field (Allerby, 2022; Alsén et al., 2022; Gabrielsson et al., 2015; Mezzich et al., 2017), and more recently in the field of suicidology (Hawton et al., 2022; Michel, 2016). This concept is not entirely new. As early as 2001, Dieserud and colleagues examined the impact of change in self-efficacy—commonly used as a proxy for measuring person-centeredness—as a mediating factor in suicidal behaviour (Dieserud et al., 2001).

Person-centred care has been described as a holistic approach that emphasizes what matters to the patient, based on a mutual understanding and shared decision-making (Barry & Edgman-Levitan, 2012; Christodoulou et al., 2016; Ekman, 2022; Ekman et al., 2011; McCormack & McCance, 2006; Mezzich et al., 2016; Morgan & Yoder, 2012). It differs from interprofessional collaboration and patient-centred coordinated activities, which involve the collaborative processes centred *around* the patient (Biringer et al., 2021; Ekman, 2022; Håkansson Eklund et al., 2019), although these processes can contribute to person-centred care delivery. While person-centeredness has been explored in other care contexts (Feldthusen et al., 2022; Nkhoma et al., 2022), and some research includes input from people with lived experience of suicide preventive interventions (Watling et al., 2022), the literature specifically addressing person-centred suicide prevention remains scarce. In 2014, Duberstein and Heisel described person-centred prevention as a humanistic approach that addresses psychosocial issues, enabling interventions that go beyond risk mitigation, thus promoting patient autonomy and self-care competence (Duberstein & Heisel, 2014). Building on previous mental health research, person-centred care promotes a holistic view of each patient, extending beyond the symptoms associated with an illness, injury, or condition (Allerby et al., 2022). Co-creation of care activities is highlighted as a vital component, integrating the perspectives of both patients and healthcare professionals to create a care plan that respects and acknowledges both viewpoints (Allerby et al., 2022). Transparency and openness in communication and decision-making are essential values during the co-creative process (Michel, 2016). Clinicians can enhance person-centeredness by actively listening, nurturing the patient’s skills, and demonstrating genuine interest in their resources and future aspirations (Hawton et al., 2022).

Although person-centred care is likely appropriate for individuals experiencing suicidal thoughts and behaviours (Hawton et al., 2022), there is limited understanding of how person-centeredness is defined and perceived by those with lived experience of suicidality. As mentioned above, most available research focuses on generic mental healthcare (Gabrielsson et al., 2015; G. P. Smith & Williams, 2016; Wärdig et al., 2021), leaving information on the prerequisites for implementing person-centred suicide prevention limited. Furthermore, there is insufficient knowledge about which healthcare practices facilitate or impede person-centeredness throughout the suicide prevention process. Additionally, specific person-centred elements required across different suicide prevention contexts remain underexplored. To address these gaps, this in-depth interview study was conducted to explore the lived experiences of care among persons with suicidal issues.

### Aim

To inform the enhancement of person-centeredness in suicide preventive health care, this study explores the lived experiences of individuals who sought primary or secondary healthcare in Sweden during suicidal crises.

## Methods

### Design

The data for this study comprises transcribed in-depth semi-structured individual interviews with persons who sought help for suicidal issues within primary or secondary healthcare settings. In-depth interviews are well-suited for phenomenological hermeneutical analysis since this approach focuses on understanding human experiences through detailed, rich descriptions, making this an ideal method for capturing lived experiences (Lindseth & Norberg, 2004, 2022). The reporting of this qualitative study followed the COREQ (Consolidated Criteria for Reporting Qualitative Research) guidelines to enhance methodological clarity (SUPPLEMENT A).

### Participants and procedures

The participants were contacted through the non-governmental organizations (NGOs) Suicide Zero (SZ) and the Swedish Partnership for Mental Health (NSPH). Both are NGOs for patients, users, and informal caregivers in the mental health field. The NGOs shared information about the study on social media and through their websites. A total of 34 people reached out to us during the enrolment phase and were given written information about the study, along with the opportunity to schedule an interview. Five did not respond, and one withdrew due to illness. This yielded a total of 28 individuals, from 10 of Sweden’s 21 regions.

In addition to the written information, all participants were provided with oral information about the study and completed a written consent form before being enrolled. They could choose to meet either in person or via an encrypted online platform. In-person meetings were held at a neutral location chosen by the participant. Participants were informed of their right to cancel meetings, take breaks during the interviews, and to book multiple meetings (*n* = 1). They were also informed of their right to withdraw from the study and have their material destructed at any time.

The interviews were held by the first author (MR) in accordance with a semi-structured interview guide (SUPPLEMENT B). In addition, all participants were asked to complete a questionnaire (SUPPLEMENT C) for reporting of demographic and clinical profile (Table I). Given the sensitive topic, the role of the interviewer was to facilitate the narrative and help the participants give their view on the topics. None of the participants had a prior relationship with the interviewer. Each interview started with an open question concerning how the participants themselves defined “co-creation of care and person-centeredness in suicide prevention”, followed by questions about how they had experienced the aspects of the phenomenon in various healthcare settings. Follow-up questions included “could you please specify?”, “could you recall any instances when you experienced the opposite?”, and “could you elaborate a bit more?” Each interview lasted between 51 minutes and 2 hours, 9 minutes.

**Table I.** Description of participants.

	n
**Gender**	
Men	5
Women	18
Other	2
Don’t want to answer	1
Missing data	2
**Age**	
18–29	13
30–44	10
45–54	4
Missing data	1
**Living situation**	
Single-person household	12
Living with partner	3
Living with relatives	10
Co-habitation with non-relatives	2
Missing data	1
**Occupation**	
Employed	8
On sick leave	8
Student	10
None	1
Missing data	1
**Place of birth**	
Sweden	23
Other European country	3
Non-European country	1
Missing	1
**Diagnosis**	
Single	7
Two diagnoses	5
Three diagnoses and above	15
Missing data	1
**Types of diagnoses**	
Bipolar disorder	2
Burnout syndrome	6
Depression/anxiety	23
Eating disorders	6
Neurodevelopmental disorders	12
Personality disorder and self-destructive behaviour	8
Post traumatic stress disorder	3
Psychosis	2
Substance use disorder	2
Missing data	1
**Main care setting**	
Primary care	2
Psychiatry	19
Multiple caregivers	6
Missing data	1
**Length of care**	
<1 year	2
1–5 years	10
>5 years	15
Missing data	1

### Qualitative data analysis - interpretation of the narratives

A phenomenological hermeneutical approach, as described by Lindseth and Norberg (Lindseth & Norberg, 2004), was used to interpret the data. This method was chosen for its suitability in exploring lived experiences and uncovering the meaning embedded in participants’ narratives, allowing for both openness to individual perspectives and interpretive depth within their broader life context. The hermeneutical component of this analysis involved interpreting the meanings of these experiences through an iterative process, in which the researchers moved back and forth between the individual parts and the whole to achieve a deeper understanding. An example of the condensation process is presented in Table II. The method includes three steps: first, a naïve understanding is developed as the researchers explore all possible meanings of the narratives. Next, a structural analysis is conducted. Finally, themes and excerpts are interpreted in relation to the whole, including existing literature and theoretical models. The goal is to elucidate the lifeworld present in each narrative, thereby capturing the essence of the experience (Lindseth & Norberg, 2004).

**Table II.** Description of the qualitative analysis process from meaning unit to theme.

Transcribed meaning unit	Naïve reading	Condensation	Theme
*“I think, for me, it’s been like this: during periods when I’ve been feeling better or well, I’ve written a plan about what’s needed when I’m at my worst or starting to feel unwell again. Relatives have been included in that plan. Then, when things get worse, we can refer back to the plan—both the healthcare team and my relatives can say, ‘This is what we agreed on.’ I think that’s really helpful because, in those moments, you’re not always super open to involving your relatives in the care.”*	Personal experience with managing mental health during periods of recovery and relapse. Description of how the participant, relatives and the healthcare team prepare for difficult times by creating a healthcare plan when the participant is feeling better. This plan includes input from relatives and healthcare providers, and becomes a reference point when their condition worsens. The approach emphasizes the importance of having an agreed-upon strategy in place, as it can help navigate moments when the individual may not feel open or capable of actively engaging with their care or involving their relatives. It highlights proactive planning and collaboration as tools for better mental health management.	The participant, relatives, and healthcare team create a healthcare plan during more stable periods, serving as a reference during downturns. This ensures support when the participant struggles to engage, highlighting the value of preparation and teamwork in mental health management.	Hoping for the best, while preparing for the worst

The interviews were transcribed verbatim, after which the authors convened to review a sample of the transcripts and develop initial insights on content. The text was then thoroughly read by the first and last author (MR and LA) to form a naïve, open-minded understanding, with the aim of exploring all possible meanings while reflecting on potential preconceptions. The interpretation process was iterative, beginning with an inductive approach to meaning formation. Subsequently, excerpts were compared and analysed in relation to the entire text, cross-referenced with other interviews, and considered alongside existing literature to deepen contextual understanding. This methodical progression aligns with the phenomenological hermeneutical framework guiding the study. The next step involved dividing the text into meaning units, which were then condensed and abstracted using Nvivo14. Both explicit communication and latent meanings were interpreted and included. These units were compared with the initial understanding for validation. To illustrate the themes, quotes were translated into English, with slight modifications made to improve readability and ensure anonymization by removing or generalizing identifying details like dates, names, and places. The quotes were numbered according to the order in which they appear in the text. Finally, the themes were reflected upon in relation to each other and to the existing literature.

### Ethical considerations

Informed consent was obtained from all participants, and their confidentiality was safeguarded at every stage. No identifiable information was included in the final report. The interviews followed an ethical protocol based on the World Medical Association Declaration of Helsinki (World Medical A, 2013) and were conducted in accordance with the approval granted by the Swedish Ethical Review Authority (2023–02180–01). Additional time was set aside before and after each session to offer support if necessary. Participants were also encouraged to contact MR for any further information or support needed after the interviews.

### Data security management

Audio files were recorded using a digital recorder. About half of the material was transcribed by MR. The other half was transcribed by an authorized transcribing company. All sensitive data were stored on a digital platform with a security classification of “highly sensitive”.

## Findings

**Figure 1.**
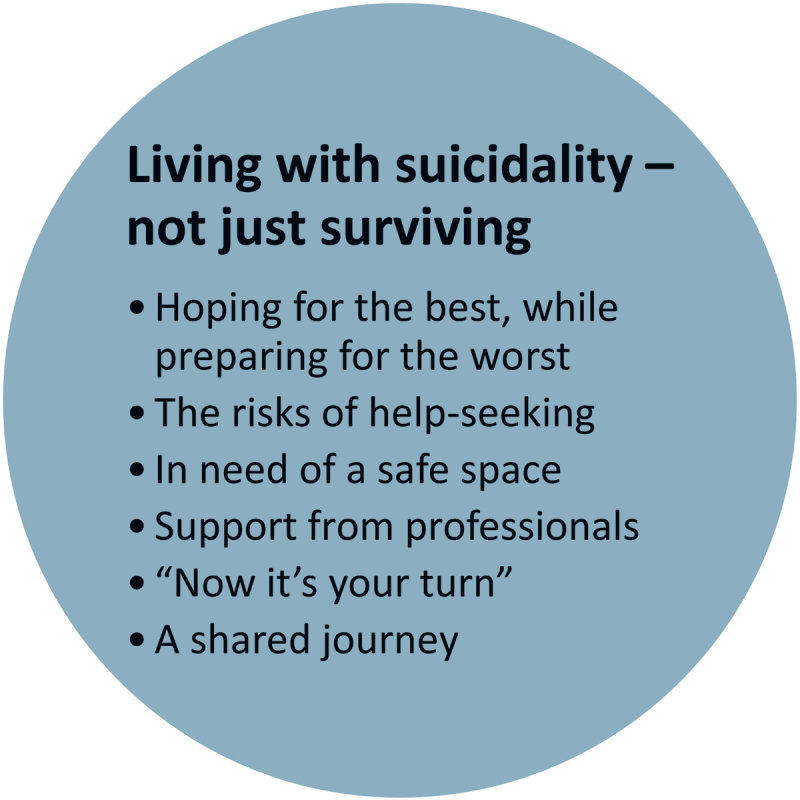
First naïve reading and main themes.

The analysis produced six themes (Figure 1). All themes are interconnected and should be understood as representing the healthcare experiences of individuals living with suicidality. While many of the insights apply throughout the suicidal process, the themes are organized to reflect experiences with healthcare at different stages, including the initial signs of worsening, seeking help, inpatient care, and post-discharge care.

### First, naïve understanding

#### Living with suicidality—not just surviving

The participants expressed that suicidality was not something you survived just once, but a recurring state you had to live with. They described living with suicidal thoughts and behaviours as a situation in which crucial aspects of their personhood were at risk. As a result, they expressed a need to restore their sense of self. Viewed through this lens, effective person-centred suicide prevention care was characterized by the participants as being reliable and supportive, and focused on reducing the consequences of suicidality while also safeguarding personal well-being.

It really feels like there’s a wall between these two realities, and it’s so difficult to reach the normal one on your own. So, it’s really helpful if someone can bring it back to you for a while and offer a bit of it. (P1)

Looking back, several participants had encountered care that they perceived as non-person-centred, delivered by professionals who viewed suicidality solely as a biomedical symptom that was either static, or progressed from low to elevated risk in a predictable pattern. In contrast, staff who demonstrated an understanding of the complex interplay between suicidal thoughts and behaviours and mental health care were highly valued. Components of person-centred care included recognizing the patient’s struggle, actively listening to their perspective, and integrating overarching goals related to their personal identity into the healthcare process. During frequent episodes of intense suicidality, the professionals shielded the patients from harmful activities and assisted them in reconstructing their sense of self, finding a new way forward. Participants stressed the importance of transparent and bureaucratically effective systems, minimizing delays and unnecessary examinations. Participants felt that too often, the focus seemed to be on maintaining routines or accommodating organizational structures rather than addressing their actual needs.

### Structural analysis

#### Hoping for the best, while preparing for the worst

Living with suicidality was perceived as an ongoing struggle, marked by fluctuations between feeling disconnected from and rediscovering one’s sense of self. This state of “in-betweenness” posed two significant threats to personhood. The first threat stemmed from the illness itself—not only the risk to life but also the loss of one’s sense of identity, as if suddenly confronting a stranger. This loss profoundly affected social, relational, and economic aspects of life, making it difficult to sustain daily activities. The second threat involved being perceived by others as merely a reflection of one’s diagnosis or having personal stories reduced to fit existing, coarse templates for assessment. Both threats were perceived as equally harmful to long-term recovery.

The participants emphasized both their need to be involved in their own care and their accumulated knowledge of how their suicidal issues manifested at different stages. Over time, they had developed strategies to avoid triggers, recognize early warning signs, manage milder states, and, when none of these measures proved effective, seek help. As suicidality-related ambivalence increased, the likelihood of seeking voluntary care decreased. Therefore, participants emphasized the critical importance of alerting others before it was too late.

I try to get them on board so we can work together because I can’t really handle it alone. But it’s always the same response: ‘Oh, hang in there, so far you’re doing fine!’ So I struggle on, but it doesn’t work out, and eventually, I feel so bad that I no longer want help. Either it ends in a suicide attempt, or a relative calls the psychiatric emergency room, and I get admitted that way. Every time it results in involuntary commitment and unfortunately, quite long hospital stays because I’ve sunk so deep into the depressive episode. And nine out of ten times, I hear, ‘Why didn’t you ask for help earlier? (P2)

The fluctuations in the condition led to drastic shifts of healthcare foci: calm periods were optimal for networking, healthcare plan revisions and recovery-oriented objectives. In addition to having defined safety measures together with care staff, continuity with a designated healthcare team was considered a valued resource. Participants who took part in planning their own care felt more equipped to manage self-care in the initial stages and were more confident that, as their condition worsened, they could address the challenge together with the healthcare team. Only a few of the participants reported having experience of such a team. Rather than taking the time to understand the patient’s perspective and integrating it with professional expertise, many felt they were treated according to an underlying protocol. Tick box questionnaires, as opposed to clinical experience seemed to guide the evaluation. Participants felt that it was difficult to receive help for the issues they themselves prioritized. Instead, they were offered whatever was available at that particular care centre, lengthy examinations for yet undiscovered diagnoses or rejection of the requested care and referral to another caregiver. Those with multiple diagnoses often faced fragmented care spread across different providers, leading to a sense of falling through the cracks due to unclear boundaries of responsibility.

#### The risks of help-seeking

In periods with intense suicidal spikes, in-patient care was considered the only realistic option. The participants described this state as existing in the twilight zone, wedged between two realities split apart by the illness spiralling further out of control. Being met with dignity, acknowledgement of the person’s self-caring efforts so far and recognition of how exhausting this had been, were all appreciated as expressions of person-centeredness. Also, the ability to recognize the person underneath the bell jar and help them reconnect was valued as person-centred. After being suicidal for a while, the suicidality tended to drain the participants of the ability to accept help.

I do appreciate it when I’m feeling a bit better and more in touch with my cognitive functions, but at my lowest, I often felt I needed them to take charge and make decisions for me. I remember being asked about admission once, and I couldn’t answer at that moment. Looking back, I see that I did need it, but at the time, I felt like I didn’t have the right to ask for anything. (P3)

The participants expressed that seeking help for suicidality carried a sense of jeopardy. Early or repeated help-seeking could lead to being rejected as merely attention-seeking, while those who delayed or avoided seeking help faced not only the dangers of untreated illness but also the potential for drastic interventions. In some cases, participants felt that involuntary care was used as a lingering threat, which could suddenly materialize if they did not comply.

If you already feel miserable and don’t want to exist, then sitting there with a threat hanging over your head—that you might get locked up if the doctor is in a bad mood—means you don’t seek help. (P4)

Because of the tradeoff between exacerbation of their condition and the risk of being dismissed or treated disrespectfully, participants described carefully weighing the pros and cons before deciding to seek help from healthcare services. When they finally did seek help, suicide preventive care was often funnelled down to a single question: “Is it time for hospitalization?” This reductionist approach was widespread among service providers and differed greatly from the participants’ own vision of adequate care, which emphasized low-intensity treatment and targeted support throughout the entire process. Instead, many felt they were confronted with an all-or-nothing scenario, where they were scrutinized for signs that spoke for or against inpatient care, which hindered co-creation of care. The participants were either functioning too well to receive any help, or too ill to make their own care decisions. To get help, they had to present with just the right number of symptoms, at the right point in time.

While participants who had a well-established relationship with their healthcare team were able to mobilize quickly when symptoms worsened, those who lacked such a connection felt they were left on their own. As their illness progressed, many described how they lost touch with the idea of seeking professional help as their illness progressed; they began to redirect their focus on finding other ways to escape their despair, only to aggravate the situation further.

#### In need of a safe space

The participants, especially those with experience of involuntary care, discussed the difference between external security and feeling truly safe. They emphasized how both the environment and the people around them influenced their perception of safety. The concept of safe spaces and persons was extended to include in- and outpatient units, emergency rooms, and transports between care facilities, where routine security measures could paradoxically reinforce feelings of being labelled as “mad” or “dangerous.” Repeated relocations for examinations and treatment procedures evoked a sense of displacement:

While you’re being transferred, you might be thinking, ‘Should I run? Should I run? Should I run? I’ll run later.’ It stirs up a lot of thoughts. And then passing through—sometimes it’s these dark tunnels they roll you through, and it feels like you’re just traveling from one light to the next. You’re being wheeled through, and you don’t even know where you are. That uncertainty is pretty scary. (P5)

Safe environments were seen as those that not only addressed external security but promoted reflection, communication, and connection. Sensory stimulation, including daylight, live plants, and the smell of food, was highly valued. Additionally, spaces that encouraged interaction with other patients were appreciated. Participants emphasized that it was crucial to not only encourage calming activities like reading, watching TV, or doing puzzles, but also to offer opportunities to exercise, to encourage creativity and to teach alternative ways to manage anxiety in a healthy manner while limiting access to unhealthy habits (e.g., vomiting and cutting oneself).

#### Support from professionals

Being acutely suicidal was described as if life itself was out of order. Reconnecting with life required staff to be physically and emotionally present, listening to patients and supporting them through their psychological turmoil. Receiving support from professionals with personal experience of mental health issues was particularly appreciated:

He said he had stood on rooftops thinking, ‘No, I’ll do it tomorrow’. And then every day, he’d think, ‘No, I’ll do it tomorrow’. So, sharing something like that—sharing his own experiences—really made a difference. It sparked thoughts in my mind, like ‘maybe I should put it off until tomorrow, too. (P6)

Since isolation, self-criticism, and paranoia could lead patients to withdraw, participants emphasized the importance of staff taking the initiative to engage with patients from the start, rather than waiting for the patients to reach out. It was also important for staff to understand the patient’s behaviours as coping mechanisms. For instance, struggling with hallucinations could prompt actions that might seem irrational to others but served logical purposes for the patient, such as touching others to confirm they were “real” or discarding objects to prevent future self-harm in response to imperative auditory cues.

In discussions on how professionals can enhance safety, engaged listening was frequently mentioned. This differed from providing solutions, which made the participants feel that professionals were emotionally unable to connect with them. Additionally, being realistic and not pretending that the suicidality would suddenly vanish was beneficial:

“I had just returned to the ward. We were sitting and talking for a while, and he said, ‘Yeah, life is a constant struggle. But that’s life.’ […] Even now, when I feel more okay and a bit more stable, I really appreciate that he said that. It wasn’t some ‘Oh, keep fighting!’ kind of comment. Instead, it was more like, ‘Yeah, life is going to suck, but it can also be pretty nice sometimes!’” (P2)

In addition to maintaining updated skills in mental health care, participants also emphasized the need for care professionals to be proficient in detecting, identifying, and treating issues arising from somatic conditions. While staffing shortages clearly affected the sense of accessibility, having too many, or uncoordinated staff members was also problematic. In some cases, being assessed in a room crowded by doctors, nurses, and their students made participants feel as though they were being interrogated. When multiple caregivers had diverging ideas on which strategy to follow, it created uncertainty about whom to trust.

#### “Now it’s your turn”

The discharge process was viewed as a particularly vulnerable period, as energy levels were naturally low, making it challenging to immediately keep up with the demands of everyday life at home. Ideally, the transition from inpatient to outpatient care should be seamless, with both services overlapping. Participants who had experienced visits from their outpatient team during inpatient care mentioned this as particularly appreciated, as it reinforced the sense of continuous support, reminded them of their ordinary life, and helped them prepare for the time after discharge.

Going forward, the most important factor was having an intact confidence in the healthcare team’s ability to care for one’s well-being. Patients who had been invited to co-create their care cited instances in which the healthcare team made efforts to involve them in treatment planning and evaluation as examples.

“’Now it’s your turn to do your part.’ There was a bit of a push, but also an acknowledgment that I was capable—they knew what I could do. They gave me a discharge date, and I was included in the discussion about when it made sense for me to leave. There was going to be an end to this. And it turned out so well. I had a say in what would happen next, and it was the calmest, most undramatic hospital stay I’ve ever had. […] And really, nothing had changed — I was the same person. But I was with people who knew me, who set some expectations for me, but who I could also trust when they said I was competent”. (P7)

The post-discharge goal was to regain enough stability to move on while having a backup plan in place, should the suicidality re-emerge. Achieving stability required practicing routines and holding meetings with involved networks well before discharge to minimize potential gaps. To safeguard person-centred principles, the participants described the most effective approach as adopting a “two experts, one plan” strategy. They particularly highlighted the importance of being actively involved in setting goals and stressed that finding a balance between risk and ambition was especially important. Focusing solely on minimizing risk often diminished other significant values in life, whereas utilizing the patient’s own capabilities could support the recovery process. Negative experiences of being excluded from decision-making could contribute to reluctance in seeking help for future health issues, both mental health issues and other.

#### A shared journey

Throughout the interviews, participants highlighted the importance of peers. Patients shared and discussed their health journeys with each other, fostering new relationships. While some connections were brief, limited to their time on the ward, others endured beyond discharge. This sense of community shaped their future choices and expectations of healthcare. Peers were not only valuable sources of information but also provided crucial support, thus adding to the sense of safety.

It’s that feeling of not being alone, I think. And the way people are so good at giving advice and supporting each other. There’s something therapeutic about it—talking to others. It gives you new perspectives. Plus, when you’re the one giving advice, you start thinking, ‘Maybe I should take my own advice and do what I just told them to do.’ (P8)

The involvement of relatives in patient care elicited mixed reactions among participants. Some viewed it as a natural and supportive element, particularly in the aftermath of inpatient care, while others highlighted potential risks to patient integrity due to relational tensions. Participants who were minors at the onset of suicidality had ambivalent feelings about the emphasis on family life. They noted that the focus on familial involvement sometimes overlooked problematic dynamics, such as parents taking on roles typically handled by professionals, e.g., extended monitoring. This role shift often caused children to lose their sense of their parents as “just parents”, blurring the lines between being informal caregivers and professionals, and further complicating their relationships with both their parents and siblings. Overall, many participants recommended that healthcare teams ask for the involvement of a “trusted person” in mental healthcare planning, rather than defaulting to asking for a “relative.”

The participants were generally positive about healthcare providing support to their relatives. However, when it came to sharing more private matters, such as psychotherapeutic content, they were more hesitant. The same caution applied to sharing medical records with other professional caregivers. Some participants believed that sharing this information could help streamline the process, particularly given the many obstacles they already faced, and were relieved not to have to act as messengers between caregivers. Conversely, others had negative experiences, feeling that sharing information led to them being singled out and treated differently, as if they had been labelled mentally unstable.

## Discussion—interpretation of the whole

In accordance with the phenomenological hermeneutical methodology, the third step of the analysis includes interpreting the findings in relation to existing literature and theoretical models, as well as discussing the parts in relation to the whole (Lindseth & Norberg, 2004).

The findings reveal that participants consider suicidality not as a one-time event but as a recurring state that threatens essential aspects of personhood. From their perspective, person-centred care was characterized as reliable, delivered by professionals who understood and advocated for the patient’s life goals while also safeguarding the well-being of their vulnerable self. Participants emphasized the importance of involving them, preferably at an early stage. They described stable periods as times when their ability and motivation to actively engage in their care were at their peak. Throughout the interviews, they stressed that co-creating care strengthened their sense of owning their own history. Those who had co-created their care reported developing self-care strategies that helped prolong these stable periods and expressed feeling confident about quickly accessing help if their condition worsened.

During periods with frequent suicidal impulses or heightened risky behaviour, participants reported being less able to collaborate—some isolated themselves, neglected self-care, or lost hope in receiving help. In such moments, it was crucial to have safety mechanisms in place, such as a safety plan or a trusted person who could advocate as a substitute or “stand-in” self. At their lowest points, participants emphasized the importance of accessing people and places that not only prevented self-harm but also helped them reconnect with life. Once their suicidality decreased, participants expressed a desire to regain greater control. In these situations, being excluded from decision-making was described as hindering recovery.

According to the participants, the preferred method to prevent worsening of their condition was maintaining contact with a designated healthcare team. During stable periods, when suicidal impulses were infrequent, there were opportunities to tailor care to align with the patient’s daily life and future goals and prepare for times when suicidality becomes more pronounced. These preparations could involve developing formal safety plans to manage escalating suicidal behaviour, but equally important was the time spent exploring what the patients perceive as important in life, and how they want to be cared for during periods when their sense of agency is dominated by suicidal ideation. This approach allowed care to be adjusted according to the patients’ current needs and context, harmonizing with personal priorities and with the goal of regaining autonomy and reclaiming their ordinary life at home. Such a perspective required shifting the focus of suicide preventive care from emergency care units to outpatient settings and redefining the goal—from identifying high-risk individuals to understanding and supporting what each patient needs in order to sustain a meaningful life within their existing contexts. Rather than emphasizing distal, static risk factors, this approach prioritizes gaining time over precisely timing interventions—supporting activities that help prevent the transition from suicidal ideation to action. This shift recognizes the inherently dynamic nature of suicidality and highlights the importance of attending to proximal, situational risk factors. Such an orientation aligns with Rudd’s Fluid Vulnerability Theory, which emphasizes the interplay between enduring vulnerabilities—both inherited and acquired—and the situational contexts that may activate them (Rudd & Ellis, 2006). Central to both this theory and person-centred suicide prevention is the guiding question: *What matters, to whom, at what time? —*along with a commitment to identifying and strengthening sources of resilience through a collaborative process involving patients, professionals, and, where appropriate, relatives.

A simple analogy may help illustrate the need for a multidimensional approach, which lies at the core of person-centred suicide prevention. Protecting a home from fire involves several layers of prevention. The construction of the house plays a foundational role; functional fire alarms, accessible fire extinguishers, and multiple emergency exits are also essential. Yet ultimately, what may matter most is the occupant’s readiness—knowing how to identify and respond to fire-related hazards, whether by extinguishing the fire themselves, calling emergency services, or evacuating safely. Similarly, effective suicide prevention must move beyond time-sensitive, acute assessments to encompass adaptive preparedness, situational awareness, and personalized support strategies. From a healthcare perspective, this is not entirely new; in other disciplines, such as maternity care, cohesive care pathways are already in use, spanning multiple services, with a range of goals to address patients’ varying needs for both preventive and acute interventions (Magnusson Österberg, 2022; Rohman, 1177.se). In this way, person-centred care can be seen as an overarching framework for future actions, rather than a detailed schedule or algorithm.

Participants with negative healthcare experiences associated seeking help not only with positive feelings but also with a sense of risk. Those who felt dismissed or perceived healthcare professionals prioritizing organizational needs over their own saw this as conflicting with person-centred care. In several cases, this led them to seek alternative solutions, or more frequently, to experience worsening symptoms until they became acutely ill. Those without a dedicated care team struggled to comply with what they believed was expected: seeking care only for conditions they felt were severe enough for intervention. This “just-in-time healthcare” was poorly aligned with their needs, reflecting only what they believed was available. While they temporarily accepted these suboptimal conditions, it eroded their long-term trust in the healthcare system’s ability to address their suicidality. Over time, this lack of trust could lead to avoiding future care altogether—a point that has also been highlighted by previous studies (Hom et al., 2015; Waern et al., 2016).

A recent study by Hagen et al (Hagen et al., 2024), which examined the roles of therapists and patients dealing with suicidal issues in a Norwegian emergency department, found that an excessive focus solely on crisis intervention shaped staff behaviour as well, and led to a prioritization of rapid patient discharge over the patient’s well-being. Our findings aligns with their conclusions, underscoring the benefits of initiating care earlier and maintaining care relationships over time to prevent a scenario in which progressing suicidality influences the patient’s behaviour, narrowing the range of possible interventions to predominately acute measures. Studies involving individuals with borderline personality disorder—a condition often characterized by self-destructive behaviours—also reveal several similarities. For instance, a recent study by Liljedahl et al. (Liljedahl et al., 2023) highlights that recovery is a lengthy process and that goal-setting must go beyond symptom remission to encompass the achievement of “a life worth living”.

The findings of our study suggest that individuals with suicidal behaviour may associate person-centred care with both collaborative and more direct decision-making, depending on where they are in the process. Recognition of the patient as a person with the inherent capacity for change was considered a central aspect of person-centred care. As discussed by Ekman (Ekman, 2022), healthcare teams play a crucial role in bridging the gap between patients’ capabilities and goal-achievement, providing the resources needed to turn these abilities into action. As discussed above, balancing non-judgemental, empathetic listening with taking action when necessary was emphasized as particularly important. Considering previous research on the interface between healthcare and suicidal individuals, it appears as though all parties involved—patients (Dubruel et al., 2024; Espeland et al., 2023; Ljungberg et al., 2015; Montross Thomas et al., 2014; Talseth et al., 1999), professionals (Biringer et al., 2021; Omerov & Bullington, 2021; Omerov et al., 2020; Waern et al., 2016), and family members (Castelli Dransart & Guerry, 2017)—value a strong working alliance, in which different perspectives are integrated in the healthcare plan. However, this research also indicates that professionals often feel uncertain about how to strike this balance (Waern et al., 2016).

From an abstract point of view, the exchange between healthcare and the patient can be regarded as a negotiation between two main parties, where future opportunities are at stake. Systems with fewer affordances—defined as the possibilities offered to an individual by the environment (Gibson, 2015; Norman & Norman, 2013), create predictability, provided that the involved parties and the setting in which they interact behave in a foreseeable manner. This can be likened to a chessboard, where each player has a limited number of possible moves. However, in healthcare settings, cases rarely follow such a structured pattern. As a result, systems that offer only a “small number of possible moves”, especially those that reduces the role of the patient to being a “helpless receiver” throughout the process, are likely to be perceived as rigid and may be seen as inadequately aligned with patients’ perceived needs. Examples of how interactional components influence the outcome of a meeting can be seen in conversational analyses of suicide-related emergency calls. Even in these high-pressure scenarios, the meaning of suicidality is continuously reframed in collaboration between the suicidal person and the responder (Iversen, 2021). Interestingly, a responder who offers a more protocol-driven conversation risks disengaging the individual, whereas one more attuned to the emotional needs of the person may ultimately gain crucial information needed to dispatch emergency services (Kevoe-Feldman & Iversen, 2022). Similarly, emergency responders negotiating with individuals at suicide hotspots might have a better chance of challenging the perceived necessity of suicide if they first seek to understand the person’s perspective on their situation (Sikveland et al., 2019). Finally, using a predetermined, gateway approach to inquire about suicidality has been shown to steer patients towards non-disclosure, making it less effective in eliciting information about suicidal ideation (McCabe et al., 2017; O’Reilly et al., 2016). Although the contexts differ significantly in terms of time frame and prior knowledge, offering numerous affordances—such as investing in trustworthiness by listening and responding based on the patient’s communication—appears to be effective when attempting to help individuals with suicidal behaviour. In the long term this strategy can become crucial in future situations when a person is contemplating whether seeking healthcare—any type of healthcare—is worthwhile.

The calculation of possible outcomes of help-seeking require both knowledge about how the system works and the skills to operate in it, leading some of the participants to believe that suicide preventive care in its current form is only available to patients who are capable, well-behaved and have no other factors that could be considered compromising to their case. This finding highlights another issue, similar to the “menu perspective” used by Desai et al. (2021) to discuss how the organization’s drive for success leads to a preference for “ideal patients”—those whose issues align neatly with the available services. These ideal patients are expected to be active and cooperative, agree that solutions lie solely within the service, share the provider’s views on goal achievement, have minimal cultural beliefs influencing their care, and be receptive to treatment (Desai et al., 2021). Given that Swedish mental healthcare is based on a model in which those who spend the most time with patients are not the ones making decisions (Gabrielsson et al., 2014), where a dilemma is perceived between fostering a strong therapeutic relationship and ensuring safety (Waern et al., 2016), and where many professionals fear that shared decision-making could lead to negative outcomes (El-Alti et al., 2022), there is reason to believe that patients with suicidality are, in fact, regarded as “unideal patients” for reasons unrelated to their capabilities. Furthermore, while examples of individuals who recovered after being seen as capable partners in care highlight the impact of person-centred approaches, equally important are the less explicit narratives: people expressing a desire merely to be recognized as credible sources of their own stories and as individuals with a genuine interest in caring for their well-being, despite struggling with suicidality. To avoid favouring only “ideal patients,” person-centred suicide prevention must address the diverse needs of all patients throughout the suicidal process.

The concept of *homo capax*, the capable individual, has traditionally been a focal point in the research field of person-centred care (Ricœur & Backelin, 2011). However, this study highlights that the complex nature of suicidality often complicates this approach. Given the long-term suffering faced by many individuals with suicidality, suicide preventive care should not only focus on immediate interventions for those who can express their needs but also offer consistent support for individuals who at times may struggle to communicate. Collaborating with interest groups to discuss suicide prevention efforts is essential for fostering a sustainable person-centred approach in mental health care. This can be achieved by involving individuals with lived experience of suicidality, both those with personal experience as well as those close to the suicidal person, in designing suicide prevention care pathways, including communication systems, and the physical spaces patients utilize during the healthcare process.

## Conclusion

This study highlights how individuals with lived experience of suicidality understand person-centred suicide prevention as a flexible, relational, and context-sensitive process-one that extends beyond the detection of risk to include proactive, co-created strategies that foster resilience and affirm personhood. Effective prevention, from this perspective, is not a standardized algorithm but a dynamic framework attuned to what matters most to the person, at a given time, within their lived context. As suicidality is experienced as both recurring and destabilizing, care must be anticipatory, personalized, and inclusive of both collaborative and protective elements. Crucially, this approach calls for a reimagining of health systems—not as rigid structures favouring ideal patients, but as responsive environments that offer trust, continuity, and multiple points of engagement. Supporting this shift requires sustained dialogue with people who have experienced suicidality, ensuring their voices guide the development of practices that are truly person-centred and capable of holding space for both vulnerability and capability across time.

## Strengths and limitations

The study draws upon the combined strengths of a research team with varied backgrounds in organizational research, person-centred care, patient safety, and suicidology, enhancing the analytical rigour and depth of the investigation. Individuals with lived experience were actively involved throughout all stages of the study, contributing to the development of a more effective interview guide by helping ensure that relevant and meaningful questions were asked. Their involvement also enriched the interpretive process by deepening our understanding of the narratives presented. To reduce the risk of misinterpretation and maintain authenticity, the interview process emphasized the participant’s perspective. When participants posed direct questions—such as, “What is your opinion about what I just shared?”—the interviewer intentionally redirected the focus back to the participant. This approach clarified that personal opinions lay outside the scope of the research and helped minimize any potential influence on participants’ responses.

We aimed to capture a broad range of experiences from persons with lived experience of healthcare in the context of suicidality. Open recruitment through a suicide prevention organization resulted in increased interest; the organization’s dissemination of a sharable recruitment advertisement via social media likely broadened its reach beyond the members of the organization. In contrast, using hospital registries or similar sources to reach out to potential participants would be ethically questionable. Interviews were organized based on participants’ preferences, offering both in-person and online meetings. The participants’ care narratives included both primary and secondary care, covering different stages of care across various regions of Sweden. There was also diversity in the reported diagnoses and the duration of care.

However, this study also has some limitations. The recruitment strategy is prone to selection bias, with risk of disproportional representation of certain groups. The majority of the participants were women, and all were young or middle aged. Although we did not specifically inquire about this, we assume that the general health and digital literacy of all participants was high. The homogenous characteristics of the participants, combined with the structure of the Swedish healthcare system, limit the transferability of our findings to other settings. Lastly, we wish to address the interpretation process. While the preunderstanding of the research team facilitated a shared knowledge of the discussed healthcare systems, it also introduced potential bias in the way the interviewer interacted or was perceived by participants. For example, participants might have assumed shared views and tailored their responses accordingly, or hesitated to express criticism. Additionally, language and cultural differences represent further factors that could shape both participation and the data collected. To ensure trustworthiness and validity, all authors participated in the analysis, discussing possible meanings and reaching a consensus on the themes corresponding to both the presented narratives and the existing literature.

## Future research

Future research is needed to elucidate how the concepts of transparency and institutional trust can be developed and operationalized within care pathways for individuals with suicidal behaviour. Further studies would also benefit from exploring how the key elements of person-centeredness could be integrated into a healthcare model that can be tested for feasibility. Additionally, we suggest studies that include the perspectives of other stakeholders on person-centred suicide prevention, such as relatives to persons experiencing suicidality, as well as their health care professionals. Finally, we recommend in-depth studies on person-centred suicide prevention for specific subgroups to ensure broader applicability.

## Supplementary Material

Supplement C questionnaire.docx

Supplement B Interview guide.docx

SUPPLEMENT A COREQ_Checklist.docx

Manuscript with author details_clean copy.docx

## Data Availability

Data is shared upon reasonable request.

## Interviewer characteristics

At the time of this study, MR was a doctoral student at the Centre for Person-Centred Care (GPCC), University of Gothenburg. She holds a background in social sciences, has been practicing as a registered nurse since 2007 and as a psychiatric nurse practitioner since 2015. In 2016, she transitioned into the role of a patient safety manager, where her responsibilities include conducting root cause analyses following incidents of suicide. Her first language is Swedish.

## Patient and public involvement

This study was designed in collaboration with Suicide Zero (SZ) and The Swedish Partnership for Mental Health (NSPH). Individuals with lived experience of suicidal behaviour participated in the analytical process, contributing their insights on the material.
