# Supplementary material for: Person-centred suicide prevention: key elements from the perspective of people living with suicidality
Source: Int J Qual Stud Health Well-being. 2025 Aug 28;20(1):2549752. doi: 10.1080/17482631.2025.2549752 (PMC12395621; doi:10.1080/17482631.2025.2549752)
Supplement: Supplement C questionnaire.docx [file ZQHW_A_2549752_SM7777.docx]

# Questions for reporting the demographic profile

Please read through the following and mark the option that best applies to you. The questionnaire will only be used to report which groups of participants were included in the study. No information will be linked to individual participants. If you do not have time to complete the survey before the interview, we can fill it in verbally before starting the actual interview.

_________________________________________________________________________

Gender: Female; Male; Other; Prefer not to disclose.

Age span: 18-29; 30-44; 45-54; 55-64; 65-74; >74.

Main caregiver: Primary care, Mental health care (secondary level); Somatic health care; Other caregiver; None.

Diagnoses (select all that apply): Depression/anxiety, Bipolar disease; Psychotic conditions; Substance use syndrome; Neuropsychiatric condition; Eating disorder; Burnout syndrome; Personality Disorder; other […]; None.

Household composition: Single person-household; Living with partner; Living with partner/children/grandchildren; Living in a household where one or more individuals are not part of own family (shared housing, roommates, boarders, etc.).

Occupation: student; Employed; On parental leave; Sick leave; Disability Benefit; Other, namely […].

Place of birth: […]

First language: […]

For how many years have you experienced suicidal behavior? < 1 year; 1-5 years; > 5 years.
