# Supplementary material for: Person-centred suicide prevention: key elements from the perspective of people living with suicidality
Source: Int J Qual Stud Health Well-being. 2025 Aug 28;20(1):2549752. doi: 10.1080/17482631.2025.2549752 (PMC12395621; doi:10.1080/17482631.2025.2549752)
Supplement: Supplement B Interview guide.docx [file ZQHW_A_2549752_SM7776.docx]

**Warm welcome to the interview about person-centered suicide-preventive care.**

**Checklist:**

- Introduction.
- Please turn off your phones.
- Are consent forms completed?
- Has the demographic profile survey been submitted?
- **Read aloud:** The conversation will last approximately 60 minutes. There are some prepared topics, but I encourage you to feel free to speak openly. Before we begin, I’d like to ask a few standard questions:
  - Are you seated in a way that allows you to speak without fear that someone else might overhear what you're saying?
  - Is the sound on your phone turned off?
  - Do you have any questions about the study, its purpose, or its implementation?
  - After the interview, there will be more time to talk if you need it. Please let me know if you have any questions, need support or wish to schedule an extra meeting with me.

**Read aloud:** I will now start the recording:

- Date.
- Number of participants.
- Number of interviewers.
- I have repeated the information provided in the participant information sheet. Participation in the study is voluntary, and you may withdraw at any time without providing a reason. Before we begin the interview, I would like to ask if you consent to participate in the study.

**Questions:**

1. The focus of this study is "person-centered care/co-creation of care." What does this concept mean to you?
2. In what ways would you like to be involved in your own suicide preventive care?
3. What do you believe is the role of the healthcare professionals in the care process?
4. Based on the care you have received, what has been most helpful to you during times of suicidal crises? You are also welcome to reflect on what *could have* been helpful.
5. Previous research has explored how relatives could be involved in care to improve its quality. What are your thoughts on the involvement of relatives in the care process?
6. In your opinion, what other stakeholders should be included in the care process?
7. What is your experience of situations where multiple care providers have been involved?
8. In what ways could technological solutions (such as interactive care plans, communication via digital platforms [e.g. 1177], or mobile applications) facilitate or complicate communication between patients, relatives, other stakeholders, and healthcare providers?
9. What information do you feel comfortable sharing? How would you prefer to share it, and who should have access to the information you share?
10. Tell me about the tools, support, and knowledge you believe a person at risk of suicide should have access to.
11. Finally, is there anything else that hasn’t been mentioned here that you feel is important to bring up? What other thoughts about "co-creating care" would you like to share?

Thank you for your participation. I will now conclude the recording.
